# Dynamic Cerebral Autoregulation Is Heterogeneous in Different Subtypes of Acute Ischemic Stroke

**DOI:** 10.1371/journal.pone.0093213

**Published:** 2014-03-26

**Authors:** Zhen-Ni Guo, Jia Liu, Yingqi Xing, Shuo Yan, Cunling Lv, Hang Jin, Yi Yang

**Affiliations:** 1 Neuroscience Center, Department of Neurology, the First Norman Bethune Hospital of Jilin University, Chang Chun, China; 2 Shenzhen Institutes of Advanced Technology, Chinese Academy of Sciences, Xueyuan Avenue, Shenzhen University Town, Shenzhen, China; 3 Center for Neurovascular ultrasound, the First Norman Bethune Hospital of Jilin University, Chang Chun, China; University Hospital-Eppendorf, Germany

## Abstract

**Background and Purpose:**

Stroke of large-artery atherosclerosis and small-artery occlusion are two main subtypes of stroke according to TOAST classification. The underlying mechanisms of how these two subtypes affect dynamic cerebral autoregulation (dCA) might be heterogeneous, resulting in varied clinical conditions and outcomes. We therefore studied the pattern of dCA in these two subtypes.

**Methods:**

Forty-one patients with acute unilateral middle cerebral artery (MCA) territory stroke (15 with ipsilateral large-artery atherosclerosis and 26 with small-artery occlusion) and 20 healthy volunteers were enrolled. Non-invasive continuous cerebral blood flow velocity and arterial blood pressure were recorded simultaneously from each subject in supine position using transcranial Doppler on MCA bilaterally and servo-controlled plethysmograph on the middle finger, respectively. Transfer function analysis was applied to derive autoregulatory parameters, gain, phase difference (PD), and slope of step response.

**Results:**

In the large-artery atherosclerosis group, PD in affected hemisphere was 42.9±18.5 degree, which is significantly lower than the unaffected hemisphere (72.4±29.9 degree, *P*<0.01), and the healthy group (*P*<0.01). However, PD is similar in the unaffected hemisphere and healthy group (*P*>0.1). In the small-artery occlusion group, PD in the affected hemisphere was similar to that in the contralateral hemisphere (33.8±17.9 vs. 32.6±21.1 degree, *P*>0.1), both sides were significantly lower than the healthy group (all *P*<0.001).The results of the slope of step response agree with the findings in PD.

**Conclusions:**

DCA in different subtypes of acute ischemic stroke is heterogeneous, which might be attributed to the varied pathologic changes of cerebral blood vessels.

## Introduction

Dynamic cerebral autoregulation (dCA) is likely affected in acute ischemic stroke[1], [2], [3]. Large-artery atherosclerosis and small-artery occlusion (lacune) are considered as the most common subtypes of acute ischemic stroke[4]. However, the underlying mechanisms of how these cerebrovascular diseases affect cerebral hemodynamics might be heterogeneous, resulting in varied clinical conditions and outcomes. The understanding of the pattern of dCA in these subtypes may help in stroke prevention, early treatment, and outcome predictions.

In large-artery stenosis, previous studies suggested that hemodynamics in ipsilateral side is altered due to the reduction of the effectiveness of vasodilation as the related vessels may have already dilated in compromise of hypoperfusion[5], [6]. The contralateral side is, however, unlikely to be affected due to the patency of the intracranial anastomoses[6] and other unknown reasons. Whereas, in small-artery occlusion, the changes of hemodynamics are more diffuse as the causes of cerebral small vessel diseases, such as hypertension, diabetes, and etc[7], [8], are normally chronic, which might affect dCA bilaterally.

We thus hypothesized that, in large-artery atherosclerosis, the dCA on the ischemic side is more likely impaired than the unaffected side, whilst in unilateral small-artery occlusion, dCA is impaired bilaterally. In the present study, we attempt to investigate this hypothesis by assessing the dCA in large-artery atherosclerosis and small-artery occlusion using transfer function analysis (TFA).

## Methods

### Participants

All participants gave their written informed consent prior to the investigation. The study protocol and written informed consent form was approved by the ethics committee of the First Norman Bethune Hospital of Jilin University. We carried out a prospective study of consecutive admissions to the Department of Neurology at the First Hospital of Jilin University during April 2013 to October 2013. Patients were included in this study if they (1) a first occurrence of acute ischemic stroke; (2) unilateral middle cerebral artery territory stroke with/without middle cerebral artery (MCA) stenosis; no other intracranial or/and extracranial major vascular stenosis/occlusion; (3) did not receive thrombolytic, interventional or surgical treatment; (4) a sufficient bilateral temporal bone window for insonation of the MCA. Patients with consciousness disorders or inability to cooperate sufficiently to complete the dCA examination, and insufficient quality of dCA signals were excluded. Also, patients were excluded if they had a history of atrial fibrillation, myocardial infarction, unstable angina, diabetes mellitus, visible leukoencephalopathy and dilated perivascular space shown in magnetic resonance imaging (MRI), impaired renal function, autonomic disturbance, or were taking any medication known to affect the cardiovascular or autonomic nervous system at the time of the study. Extracranial and intracranial artery stenosis or occlusion was diagnosed by transcranial Doppler (TCD, MultiDop X2, DWL, Sipplingen, Germany), carotid ultrasound (IU22, Phillips, Andover, Massachusetts, USA) and magnetic resonance angiography (MRA). Stenosis ≥50% of the MCA was determined by a mean velocity>100 cm/s; stenosis ≥70% of the MCA was determined by a mean velocity >120 cm/s in TCD[9], the results of MRA was used as a reference. Atrial fibrillation, myocardial infarction and/or unstable angina were excluded by cardiologists. Stroke patients were divided into two groups: large-artery atherosclerosis and small-artery occlusion according to TOAST classification[4]. The lacunar on MRI was shown as lesion with a diameter of less than 1.5 cm demonstrated. The large-artery atherosclerosis was diagnosed as infarcts greater than 1.5 cm in diameter on MRI, and a stenosis of greater than 50% in ipsilateral MCA. Patients were excluded if they are not in these two types. Each patient was diagnosed with stroke and classified by two neurologists according to clinical symptoms, MRI, MRA and TCD. Twenty medically and psychiatrically healthy volunteers were recruited as normal controls.

### Study Protocol

DCA examination was carried out during 5–10 days of symptom onset. Subjects avoided alcohol, caffeine and nicotine for at least 12 hours before the dCA examination, which were made in a quiet, dedicated research laboratory, at controlled temperature of 20–24 °C with external stimuli minimized. Subjects were asked to adopt a relaxed supine position for 10 minutes, and then the baseline blood pressure was measured at the brachial artery (automatic blood pressure monitor, Omron 711). Continuous cerebral blood flow velocity (CBFV) and arterial blood pressure (ABP) were recorded simultaneously from each subject in supine position for 10 minutes. The recorded data were then used to assess cerebral autoregulation.

The continuous ABP was measured non-invasively using servo-controlled plethysmograph (Finometer Pro, Netherlands) on the middle finger. Endtidal CO2 (EtCO2) was monitored using a capnograph (MultiDop X2, DWL, Sipplingen, Germany) with face mask attached to the nasal cannula. TCD was used to measure CBFV in MCA bilaterally at a depth of 45–60 mm. The probes were fixed with a custom made head frame.

### Data Analysis

The recorded data was processed by a personal computer using MATLAB (a suite of commercial software for data processing). Beat-to-beat alignment of the data was achieved using cross-correlation function to remove the possible time lags. A 3rd order Butterworth low-pass filter (cutoff at 0.5 Hz) was then applied as an anti-alias filter before down sampling the data to 1 Hz. The dynamic cerebral autoregulation was evaluated using TFA[10]. Transfer function between ABP and CBFV is calculated as the quotient of the cross-spectrum of the two signals and the autospectrum of ABP in frequency domain. Impulse and frequency responses are derived from TFA. In time domain, step response was calculated by convolution between impulse response and a normalized step to show how CBFV responses to a step change of ABP. A linear regression of the first 4 seconds of the step response was performed and the slope of the regression line was used to quantify how quick CBFV may return to the baseline level, where a steeper slope indicate a faster return[11]. In frequency domain, we estimated phase difference (PD), gain, and coherence function within low frequency range, 0.06–0.12 Hz, to evaluate cerebral autoregulation, where the derived parameters are considered most relevant to this hemodynamics[12]. We only used the autoregulatory parameters for the later statistical analysis if the coherence within 0.06–0.12 Hz is > 0.5.

### Statistical Analysis

The statistical program for social sciences version 17.0 (SPSS, IBM, West Grove, PA, USA) was used to analyze all data. The measurement data are expressed as the mean ± SD, and the count data are expressed as the rate (percentage). Student's *t*-test used to identify measurement data. The chi-square test and fisher exact test were used to identify count data. The level of significance was set at *P*<0.05

## Results

### Demographic information

In total, 41 patients (50.7±12.0 years; 33 males and 8 females) were enrolled in the study. Fifteen patients were the large-artery atherosclerosis group (44.7±13.1 years; 12 males and 3 females) and twenty-six patients were the small-artery occlusion (54.1±9.7 years; 21 males and 5 females). In the large-artery atherosclerosis group, seven patients (46.7%) had impairment in movement alone, two (13.3%) presented with loss of speech alone, and six (40.0%) had loss of function in both movement and speech; ten patients with MCA stenosis ≥50% and 5 patients with MCA stenosis ≥70%. In the small-artery occlusion group, fourteen patients (53.8%) were pure motor stroke, two (7.7%) were pure sensory stroke and ten (38.5%) were sensorimotor stroke. Twenty medically and psychiatrically healthy volunteers (42.2±13.7 years; 16 males and 4 females) served as controls. We did not find any significant differences in sex, heart rate and EtCO_2_ in the three groups. The patients in the small-artery occlusion group were approximately 10 years older in average than those in the healthy group (t = 3.4, *P* = 0.001) and large-artery atherosclerosis group (t = −2.6, *P* = 0.012). Simultaneously, the mean blood pressure in the small-artery occlusion group was higher than that in the healthy group (t = 2.2, *P* = 0.031). Baseline characteristics of the data are given in Table 1.

**Table 1:** Baseline characteristics.

	Large-artery atherosclerosis group (n = 15)	Small-artery occlusion group (n = 26)	Controls (n = 20)
Male	12 (80.0%)	21 (80.8%)	16 (80.0%)
Age (years)	44.7±13.1	54.1±9.7	42.2±13.7
Hypertension	7 (46.7%)	19 (73.1%)	0 (0%)
Mean blood pressure (mmHg)	90.0±17.0	101.5±19.7	89.5±16.0
Heart rate (beats/min)	71.7±6.4	71.2±8.8	74.3±7.4
EtCO_2_ (mm Hg)	36.2±2.6	37.2±2.9	36.4±2.4
NIHSS score	7.1±4.7	3.8±2.8	

### Dynamic cerebral autoregulation

**Figure 1:**
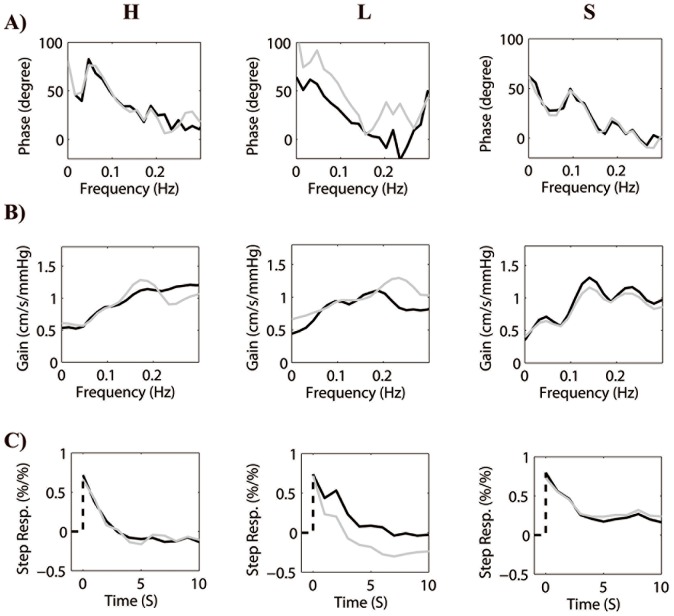
Three autoregulatory parameters, A) phase difference, B) gain, and C) step response, derived from the transfer function are plotted. H (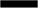: right side and 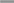: left side), L (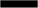: affected side and 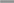: unaffected side), and S (same legends as L) denote healthy controls, large-artery atherosclerosis group, and small-artery occlusion group, respectively. The first column shows that all parameters in the healthy group from both sides indicate that autoregulation is intact. There is evident positive phase difference. The shape of high-pass filter can also be speculated. The step response, H-C), shows that cerebral blood flow velocity quickly returns to the baseline level within approximately 4 seconds if a unit step change of arterial blood pressure is induced. In the second column, the parameters changes asymmetrically. Dynamic cerebral autoregulation on the affected side is substantially reduced than the unaffected side with decreased phase difference, flattened gain, and slower return of cerebral blood flow velocity in response to a step change of arterial blood pressure. In the last column, this series of subplots show that autoregulation is worsen bilaterally when comparing with the healthy controls.

**Figure 2:**
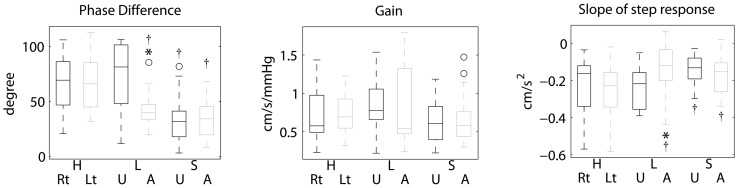
Statistical distributions of the autoregulatory parameters for each category are shown above. A) Phase difference (PD) from the affected side in the large-artery atherosclerosis group is significantly lower than the contralateral side as well the healthy group. PD from the small-artery occlusion group is bilaterally lower than those in the healthy group but there is no difference within the group. B) No statistical difference is detected using gain. C) The results of the slope of step response agree with the findings in PD. Flatter slope (the value of the slope of step response is closer to zero) is observed within the affected side in large-artery atherosclerosis group than in other conditions. The slope in the small-artery occlusion group is also flatter than the healthy controls only. H, L, and S denote the three groups as Figure 1. Rt and Lt stand for right and left side, respectively, for the healthy controls. U and A denote unaffected and affected sides, respectively, for the patients. For each box plot, the central mark is the median and the edges of the box are the 25th and 75th percentiles, the whiskers extend to the most extreme data points which are not outliers, and the outliers are plotted individually as ‘○’. ^*^denotes *P*<0.05 for comparing between two sides within the same group. **^† ^**denotes *P*<0.05 for comparing with the overall of healthy group.

Three autoregulatory parameters, gain, PD, and step response, were derived from the transfer function. The valid frequency range for dCA (PD and gain) is 0.06–0.12 Hz and the first 4 seconds of step response was used to calculate the slope. Graphical results are shown in Figure 1 and the statistical analysis of the parameters is given in Table 2 and Figure 2.

**Table 2:** Phase difference (PD), gain and slope of step response in patients and controls.

		PD (degree)	Gain (cm/s/mmHg)	Slope of step response (%/%)	
Healthy group	Left	65.8±24.1	0.72±0.26	−0.24±0.14	
	Right	67.4±24.2	0.71± 0.35	−0.23±0.15	
	Overall	66.6±23.8	0.71±0.30	−0.23±0.14	
Large-artery atherosclerosis	Affected hemisphere	42.9±18.5* **^†^**	0.81±0.53	−0.14±0.15 *†	
	Unaffected hemisphere	72.4±29.9	0.83±0.32	−0.24±0.11	
Small-artery occlusion	Affected hemisphere	33.8±17.9**^†^**	0.66±0.31	−0.17±0.09†	
	Unaffected hemisphere	32.6±21.1**^†^**	0.62±0.26	−0.15±0.08†	

*and **^†^**denote the same statistical meaning as Figure 2.

### Phase difference

In the healthy group, PD (0.06–0.12) between ABP and CBFV was 65.8±24.1 degree in the left hemisphere and 67.4±24.2 degree in the right hemisphere, which is no difference (t = 0.4, *P*>0.1, Table 2, Figure 1 and 2).The overall PD in this group was 66.6±23.8 degree.

In the large-artery atherosclerosis group, PD in affected hemisphere was 42.9±18.5 degree, which is significantly lower than the unaffected hemisphere (72.4±29.9 degree, t = −4.2, *P*<0.01), and the healthy group (t = −3.4, *P*<0.01). However, PD is similar in the unaffected hemisphere and healthy group (t = −0.74, *P*>0.1; Table 2, Figure 1 and 2).

In the small-artery occlusion group, PD in the affected hemisphere was similar to that in the contralateral hemisphere (33.8±17.9 vs. 32.6±21.1 degree, t = 0.60, *P*>0.1), both sides were significantly lower than the healthy group (all *P*<0.001, Table2, Figure 1 and 2).

### Gain

There was no difference between groups and two sides in each group (all *P*>0.05, Table 2, Figure 1 and 2).

### Step response

In the healthy group and the small-artery occlusion group, there is no significant difference between the two sides within these groups for the slopes of the step response. However, the overall slope of the healthy group (−0.23±0.14) is steeper (larger absolute value) than the affected and unaffected sides of the small-artery occlusion group (affected: −0.17±0.09, t = 2.1, *P*<0.05; unaffected: −0.15±0.08, t = 2.8, *P*<0.01; Table 2, Figure 1 and 2), respectively.

The characteristics of step response in the large-artery atherosclerosis group are asymmetric. The slope on the unaffected side is significantly steeper than the affected side (−0.24±0.11 vs.−0.14±0.15, t = 3.1, *P*<0.01; Table 2, Figure 1 and 2). Furthermore, there is no difference between the unaffected side and the healthy group (t = −0.17, *P*>0.1), whereas the slope on the affected side is flatter than the controls (t = 2.0, *P*<0.05).

## Discussion

The main findings of this study are that, in stroke patients: 1) dCA is impaired at the affected side in the patients with large-artery atherosclerosis, whereas dCA on the unaffected contralateral side remains intact; 2) the dCA is impaired bilaterally in patients with unilateral small-artery occlusion. These agree with our hypothesis and suggest that cerebral hemodynamics is varied in different stroke subtypes, indicating that the underlying mechanisms of how dCA is altered in these clinical conditions are different.

It has been reported that in the patients with severe unilateral carotid and MCA stenosis, dCA is impaired ipsilaterally when compared with the contralateral sides[5], [13], [14]. Immink *et al* found that dCA is impaired ipsilaterally on the ischemic side in the patients with unilateral large MCA territory infarcts[7]. However, these authors did not link acute ischemic stroke with artery stenosis. In this study, we considered the underlying relationship of these two and focused on this specific subtype of ischemic stroke (large-artery atherosclerosis in TOAST classification). This can eliminate the influence of other subtypes of stroke, such as cardioembolism, small artery occlusion and unexplained stroke. We thus consider that the ipsilateral change of dCA is mainly attributed to the large-artery stenosis, causing vasodilatation of the vessels in ipsilateral MCA territory in compromise of hypoperfusion. The relatively high mean value (42.9±18.5 degree, Table 2) of PD on the affected side may be contributed by collateral circulation, which is developed with progressive artery stenosis[15]. However, there is a limitation when analyzing this subtype as we cannot answer whether acute large-artery stroke additionally impairs autoregulation, because a moderate or severe upstream stenosis can cause impairment of dCA[5], [13], [14] and there was lack of control group with asymptomatic stenosis of the same degree and same collateral capacity for comparison.

We also noted that there were other studies showing that the impairment of dCA may spread to the contralateral side, which is manifested in 5–14 days after onset[2], [16], [17]. In order to achieve comparable results, we recorded the data during 5–10 days after onset and our results however did not agree with these studies. We considered the difference is mainly due to the more strict inclusion criteria, with lacunar infarction, cardioembolism and unexplained stroke excluded.

In small-artery occlusion, we included unilateral middle cerebral artery territory acute lacunar infarction and our results are consistent with Immink *et al*'s group that dCA is impaired bilaterally in lacunar stroke patients[7]. Some authors attributed this to cerebral small vessel disease[7], [8] caused by chronic diseases such as hypertension, diabetes, and etc. Though the real mechanisms of cerebral small vessel diseases remain uncertain, one of the main explanations of these pathological vascular abnormalities is arteriolosclerosis[8], [18], which increases stiffness of small vessels and decreases the systolic and diastolic function. Therefore dCA is impaired and the function of clearance of embolus decreased followed by uncontrolled CBF, eventually leading to embolism[19], [20]. It should be emphasized that cerebral small vessel disease is diffuse. The impairment of dCA in MCA may therefore represent the impairment of the whole cerebral small vessels. We thus believe that it is more likely that dCA is an important risk factor that leads to acute lacunar rather than caused by acute lacunar, because the infarcts are small, and less likely to cause global impairment. Impaired dCA can decrease the function of clearance of embolus followed by uncontrolled CBF, eventually leading to embolism and resulting in lacunar infarcts. It is however unknown if acute lacunar can aggravate dCA.

There are a few limitations of the data collection, addressed as follows. Monitoring CBFV in stenotic MCA was difficult in the patients with large-artery atherosclerosis. Data were discarded if signal-to-noise ratio is low, resulting in less data in large-artery atherosclerosis. Male subjects are more than female in our study, as prevalence of stroke in China is higher in men than women[21] and female subjects are more likely to have insufficient bilateral temporal bone window for insonation due to the low density of the temporal bone[22]. We noted that the age and ABP in small-artery occlusion are different from other groups, the differences are one of our limitations in our study. However, as previous studies suggested that small-artery occlusion is likely caused by chronic pathology from hypertension, diabetes, and etc. [7], [8], it might be difficult to control these factors which are possibly the risk factors or causes of small-artery occlusion. In addition, we selected patients with mild to moderate symptoms, as they can cooperate with the study, this may however bias the statistical analysis as possible characteristics of dCA of the severe patients might have been ignored.

## Conclusions

The current study shows that dCA in different subtypes of acute ischemic stroke is heterogeneous, which might be in association with the varied pathologic changes of cerebral blood vessels. Large artery atheroselerosis affects dCA ipsilaterally, whereas small artery disease is more likely to impair dCA globally.
